# Palliative Care: A Primary Care Pharmacist Perspective

**DOI:** 10.3390/pharmacy10040081

**Published:** 2022-07-13

**Authors:** Julia Bognar

**Affiliations:** Priory Medical Group, York YO24 3WX, UK; julia.bognar@nhs.net

**Keywords:** pharmacy, palliative and end-of-life care, medication management, polypharmacy, deprescribing, primary care

## Abstract

The overview approaches pharmacy practice in palliative care from a global viewpoint and aims to provide insight into front-line pharmacist–patient relationships by sharing case studies and personal experiences.

## Add Life to Days and Not Days to Life

The WHO defines palliative care as an approach that improves the quality of life of patients of all ages and their families who are facing problems associated with life-limiting illness. It prevents and relieves suffering, whether that be physical, psychosocial, or spiritual [1,2]. However, the impact of pharmacists’ contribution to the care of palliative patients remains less widely understood [3].

Globally, an estimated 40 million people require palliative service, and approximately 14% of these people receive it. The WHO has highlighted barriers in accessing palliative care services: strict and incredibly restricted access to morphine, lack of suitably trained health care professionals, lack of relevant policies, and the lack of integration of palliative medicine into national healthcare systems and, more importantly, into primary care systems. The WHO estimates that the global need for palliative care will continue to grow over the coming years [1,2].

The United Kingdom is ranked first in the world in the so-called “Quality-of-Death Index” because of the comprehensive national policies in palliative care, the extensive integration of palliative care into the National Health Service, the hospice movement, and the very strong community involvement on [the issue. The Marie Curie Foundation estimates that approximately 75–80% of people dying each year require palliative care, but data show that less than 50% of these receive palliative care support although the exact statistic may differ across the countries. The most common conditions needing palliative input are cancer, cardiovascular or cerebrovascular disease (including heart failure and stroke), respiratory disease, renal disease, Alzheimer’s disease and dementia, and HIV or AIDS. Over the years, it is expected that a growing percentage of patients receiving palliative care will be dementia patients [2].

Not surprisingly, pharmacists working across all sectors are seen as the hidden and often forgotten arm of any palliative care team [3,4,5]. Community pharmacies are based walking distances away from the patients, and pharmacy teams see them and their carers regularly. I have seen colleagues countless times spotting the first clues of deterioration in someone’s general health. Community pharmacists regularly consult, educate, and reassure patients and anxious family members about the use, effects, and side effects of medications. Ultimately, pharmacists are trained to respond to symptoms. Furthermore, pharmacy professionals tirelessly go above and beyond to promptly source what are many times almost unsourceable medications.

That said, during my pharmacy training all those years ago, I did not hear much about palliative care. I remember my course being incredibly science-focussed: I was up to date with the latest cancer research, the genomics, and the pharmacology of the then-available anticancer medications. I understood polypharmacy and the special pharmaceutical needs of people of all ages. However, I was told very little about what happens beyond the boundaries of evidence-based medicine: I knew nothing about what to do when all medical options to cure have been exhausted, and the disease can take over. Interestingly, I recall having attended a seminar on thanatology where we would often discuss how poorly clinicians could handle death, how there is good death and bad death, and how easily clinicians may overlook the needs of a patient with incurable disease. It was during this seminar that I first learned that one can never predict the “when” but can only plan for the “how”. However, I did not know back then how this related to the rest of my course.

Some years later, I was covering a locum shift in a rural small town community pharmacy. I still remember the exhausted, dirty looking man who walked to the counter and asked to speak to the pharmacist. He was in tears and smelt of alcohol. “I can’t sleep! I have tried all the sleeping tablets. I am fed up with going to the doctors”, he said. Then, he looked at me and added: “My wife has ovarian cancer. You don’t know what it’s like watching this beast taking her from me”. He was right. I did not know. I remember it was late. I was hungry and exhausted, too. Besides, he did not ask me about side effects or interactions. He needed something I did not have and could not offer. As a recently qualified young woman, talking to someone over twice my age about something I had never seen or experienced felt incredibly difficult, and I froze. My assistant resolved the situation: “I’ll go and put the kettle on for you two!”.

In agreement with the Gold Standard Framework guidance, patients in the UK are added onto the GPs palliative register when they are believed to be in their last 12 months of life [6]. These patients have acknowledged needs at the different stages of their palliative journey [7,8]. For instance, an average cancer patient may contact their GP at least 43 times in their last year of life. During these consults, an average of 72 prescriptions are issued, 21 of which are new drugs. In general, having palliative care needs recognised is ultimately associated with an increased number of GP contacts [7].

Pharmacists working in a primary care setting, in my view, are incredibly well-placed to support all aspects of the pharmaceutical needs of the palliative patients. Access to the full medical history and the ability to liaise with all stakeholders allow for establishing the much-desired continuity within the emerging primary-care-access models: pharmacists may act as point of contact for all medication-related concerns. This role is even more significant considering that a growing majority of palliative care patients are cared for in their homes [1,2,3,4,5,7,9,10,11].

I recall a telephone review with a 72-year-old lady who had advanced lung cancer and multiple other chronic cardiovascular and respiratory co-morbidities, including severe ischemic heart disease. She was taking several inhalers, blood pressure tablets, cholesterol tablets, an antiplatelet, and was anticoagulated with warfarin for atrial fibrillation. When I first “met” her for her medication review, she was suffering from the side effects of her radiotherapy and was treated with high-dose steroids. She lived alone. I did not even have time to introduce myself before she dropped the question: “Do I need all this?” She had just found out the previous day that her cancer was progressing; it was no longer responding to any treatments. In fact, she felt that the only tablets she needed were her blood thinner and her prednisolone. “I know I only go one way from here,” she said. As a clinician, I would have handled her medications differently, but I did not intervene. I understood that she was very keen to have her INR checked, for she appreciated the regular visits from the district nurses. She never agreed to switch to a novel anticoagulant or to stop anticoagulation for good. Her disease progressed over a few short weeks, and she had more frequent episodes of haemoptysis. A frightening experience for many but not for this patient. She learned to work out the thickness of her blood from the sputum, and she adjusted her warfarin accordingly. She was 100% right whenever her level was checked, resulting in a significantly reduced number of venous punctures for INR tests although the number of her contacts with the surgery increased.

This example demonstrates the importance of appreciating the patients’ perspectives on handling their medications. On the other hand, medication management and the medicine information needs of palliative patients and their carers are so often undermined and underappreciated by their clinical teams [3,4,5,7,9,10,11]. Latif et al. emphasise the lack of awareness of the stress and emotional burden of administering potent medicines in the domiciliary setting [9]. Tija et al. methodically identified gaps in the medication management skills and knowledge of caregivers by directly observing nurse-led consultations at the patient’s bedside [5]. All these point to the desperate need for the pharmaceutical expertise to ease the burden of medication use for patients and their carers. This gap shapes the opportunity for pharmacists and invites us to recognize and to respond more proactively to the often-unmet needs of the palliative patient population. It also underlines the requirement for active participation in direct patient care and defines a more specific role for us within the wider palliative care team.

Arguably, patients’ perceptions of their treatment change throughout the illness, even reaching a point when less importance is placed on the tablets or on their medical care. Healthcare professionals refer to this as “transition”, which often coincides with the acceptance of illness and the associated life expectancy [8,9,10,11].

Another case is that of the 65-year-old woman with a decades-long history of multiple myeloma (MM). Her treatment history included countless rounds of chemotherapies and a bone marrow transplant. In the past, she always responded to the treatment that was offered. However, when her MM came back for the last time, nothing worked. She did not have success with the first-line treatment, nor with the second- or third-line options. She became severely anaemic with an almost non-functioning bone marrow. At that point, her haematologist explained to her that “We have done everything we could”. From that day on, she was unable to sleep. She had an equally long history of depression. She was in severe pain, as the MM affected her spinal cord, her head, her shoulders, and her pelvic bones. She barely moved, ate, or drank, and she spent most of her time in bed. Yet, she declined every single intervention offered to make her feel comfortable at home. One medicine gave her bowel problems. The next made her dizzy, and so on. Her husband did not understand what was going on. This was not the woman he had known all those years who was always on top and getting everything right. I remember that it took a while to get to the bottom of her story, but the underlying reason behind her stance was this: “They (the medications) take away my sanity, and that is the last thing I have to myself!”.

I admit that I learn a great deal from these patients. Ultimately, they are people in the process of embracing their own mortality. Being touched by death is one of the most deeply transformational experiences for human beings: it teaches to adjust, to live life without future fears, and to shift the focus of the self from the “me” to the “others”. Kathryn Mannix expands on this in her book, “With the End in Mind” [12]:

“*This transformation of worldview is a spiritual transformation. (…) It enables the person to review their lives and to recognise and regret any hurt they may have caused other people and often desire to make amends.*”

Our patients are incredibly thankful for the smallest contribution from any of us, and their “thank you” is heartfelt, appreciating the good intention behind our efforts even when we feel that we did not do enough.

This latter case, in my opinion, also highlights that medication management is a complex task in palliative care [13,14,15,16,17,18,19], yet most of the available evidence remains empirical. The significance of the pharmacist’s contribution does not change even when the patient initially chooses not to be treated pharmacologically. Palliative medication optimization may not seek to tackle all aspects of polypharmacy, respecting the patients’ views on their own treatment. However, evidence shows that most patients and their clinicians, in particular the general practitioners, are in favour of rationalising medications [15,19]. Reduced tablet burden is associated with reduced risk of adverse reactions, reduced cost implications for both care providers and patients, and improved quality of life [13,14,15,16,17,18,19]. In my experience, good palliative deprescribing relies on the understanding of the patient’s values, their psychosocial context, and physical needs. Principles such as the “time to benefit” can be applied to deprescribing preventative medications, while an alternative approach is to decide not to manage co-morbidities in context of an advanced life-limiting illness [17,18].

Sailing the palliative waters, it is easy to feel out of our professional comfort zone: drug trials were never designed to include the palliative patients, and studies evaluating the effectiveness or safety of any drug or intervention are often inconclusive when applied to the palliative context. Research methodologies and evidence-collection principles in palliative medicine differ significantly to the traditional, quantitative, multi-arm, double-blind approach of the evidence-based medicine that we are so used to [20,21,22,23,24].

Nowadays, more formal training in palliative care is available for those wishing to enhance their skills and understanding of the subject. The post-graduate modules provided by universities (e.g., the University of York or the University of Newcastle) have been developed in response to the ever-growing need for the provision of the right palliative service at the right time. These courses are open and available to pharmacists. On-line learning is possible, which makes the programmes accessible to a greater number of learners, and as such, they directly contribute to improved patient access to care provided by a suitably trained clinician. The modules introduce the overarching concept of palliative medicine and focus on the assessment and management of the most common symptoms in palliative and end-of-life care as well as build confidence in recognising and managing the psychosocial and spiritual needs of patients throughout their palliative journey.

Reflecting on my own post-graduate training, I am incredibly thankful for the consultants, medics, specialist pharmacists, and nurses that I encountered during those years. Training in palliative care was a roller coaster experience for me. I saw patients at their very weakest, and I often had to learn the hard way that there is no “one fit for all” in this specialty. I remember the hours spent on working out syringe driver compatibilities just to find out that the driver never got started. I recall the countless attempts to get on top of managing the various symptoms, concluding that nothing could be done. I remember the many switches and conversions from tablets to a patch and to a syringe driver, then back to tablets, then again to a syringe driver, or from one opioid to another in hope that any of those options would better control the symptoms caused by a progressing illness. I remember the fast-track discharges, managing the discharge medications, and communicating last-minute changes, often in unsocial hours. However, I also remember the grandmothers, the grandfathers, the mums and dads, lovers, and partners who shared their stories with me: they worked, travelled, planned, dreamed, and loved. They showed me life through another lens.

I am fortunate to have visited a small rural Sub-Saharan palliative care clinic, and as such, I have first-hand experience of palliative care provided in the UK as well as in a resource-poor setting. One could analyse the shocking differences at many levels, but the very driving foundation of establishing the services in both countries is common: patients need their pains alleviated. I will always remember the teacher, in her early forties, living in a mud house, with advanced gynaecological cancer. She smiled at me when she nodded to have me participate in her appointment. I learned that she was HIV-positive, and her husband only recently gave in to allow her to take the antiretrovirals. It was the first time that I saw her type of cancer from so close. It was oozing and infiltrating her pelvic organs, blocking her from managing her very basic needs easily. The ulcer was treated with topical metronidazole powder, and her pain was managed with oral morphine. She also received oral nutritional support. Her medications were reviewed and refilled every two weeks either during a home visit or a clinic appointment. Medically, her morphine was titrated according to her needs, and she used it as prescribed. She looked pale and tired, but she had no physical pain. When I asked how she was feeling, she answered “I am failing. I am no longer a good wife”. The incredibly inspiring palliative care nurse, the Malawian-born Lucy Finch, reflected with me on this later: “You see, this is what it is about: One pain is not alleviated, you have done almost nothing! (…) Be sure that even when you have nothing, you have yourself, and that is enough to provide good palliative care”.

## Data Availability

Not applicable.
